# Exploring subjective wellbeing after birth: A qualitative deductive descriptive study

**DOI:** 10.18332/ejm/104679

**Published:** 2019-03-15

**Authors:** Fiona Alderdice, Phyl Gargan

**Affiliations:** 1 National Perinatal Epidemiology Unit, Nuffield Department of Population Health, Oxford University, Oxford, United Kingdom; 2 School of Nursing and Midwifery, Queens University Belfast, Belfast, United Kingdom

**Keywords:** wellbeing, postnatal, qualitative

## Abstract

**INTRODUCTION:**

Subjective wellbeing is made up of cognitive (life satisfaction and positive functioning) and emotional (positive and negative affect) components. The subjective wellbeing of women in the perinatal period is poorly understood compared to that experienced by the general population. The aim of this paper is to describe women’s experiences of subjective wellbeing postpartum using the European Social Survey Wellbeing module to facilitate discussion.

**METHODS:**

Nineteen women aged 18−40 years who had given birth within the past 6 months participated in two focus groups (n=9 and n=10). Participants in the focus groups were invited to complete the European Social Survey Wellbeing module and this was used as the basis for discussion.

**RESULTS:**

Women recognized that many aspects of their wellbeing were compromised after having a baby, e.g. vitality and positive functioning. Women reported that the time after birth was often challenging in terms of feeling good about themselves. Women were often tired and did not always trust their own abilities as a parent. Woman reported feeling socially isolated after giving birth and highlighted the importance of their relationships in terms of feeling valued. Changes in relationships were a source of stress and conflict. Many women reported that they did not feel engaged with their local community.

**CONCLUSIONS:**

Women perceive their subjective wellbeing to be different after birth. A better understanding of the aspects of wellbeing that may be challenged after birth could facilitate provision of tailored support for women and their family to maximize their continued good health and wellbeing.

## INTRODUCTION

The psychological wellbeing of women in the perinatal period is poorly understood compared to that experienced by the general population. Subjective wellbeing is a multidimensional concept broadly considered to have cognitive (life satisfaction and positive functioning) and emotional (positive and negative affect) components that are subjective for the individual and relative to social norms and values^1^. The term subjective wellbeing has been used interchangeably with happiness and flourishing and as a concept is not always well defined. There is no overall consensus as to all the components of subjective wellbeing; within the context of this paper a detailed model of wellbeing is used that was developed for use in the 7th round of the European Social Survey and highlights seven core components of wellbeing at the individual (emotional wellbeing, satisfying life, vitality, resilience and self-esteem, positive functioning) and social level (supportive relationships, trust, and belonging)^2^.

It has been recognised for some time that subjective wellbeing and health are closely related. Diener and Chan^3^ conducted a review of the relationship between wellbeing, health and longevity and concluded that the effect of wellbeing, while variable, is substantial and comparable to other health behaviours targeted in public health policies for healthy diet and exercise. The link to health is now acknowledged in health policy, for example the World Health Organization^1^ highlighted the need to set wellbeing targets as part of European Health 2020 policy and the Department of Health England have stated that wellbeing is a shared government objective^4^. Maternity care policy recognises that most women are well and that promoting emotional wellbeing is as important as supporting physical health^5^. While mental health problems are increasingly screened, it is important to broaden the assessment of psychological status during pregnancy in order to take action to optimize wellbeing at this time^6^.

The term ‘wellbeing’ in policy and practice, however, is often loosely defined and is not linked to research that explores the psychological construct of subjective wellbeing as outlined in this paper. Our limited knowledge comes predominantly from population-based studies using a single question on happiness and from literature on negative emotions particularly depression. Myrskylä and Margolis^7^ studied parental happiness trajectories before and after the birth of a child, using large British and German longitudinal data sets and found that happiness increased in the year before birth and through the year after birth but then decreased to before-child levels. Parity, age and education were associated with happiness responses with older or more educated parents having a particularly positive happiness response to a first birth. The first two children increased happiness response, whereas a third child did not^7^. Aassve et al.^8^ found, in a large European sample, that partners were overall happier than non-parents, however happiness was strictly related to a partner being present^8^. These cohort studies used simple one item questions to explore happiness and it is likely that parental wellbeing around the time of childbirth is complex and needs further investigation^9^.

Subjective wellbeing is a spectrum and includes an evaluation of both positive (e.g. happiness) and negative (e.g. depression) emotions. As we noted, our knowledge about wellbeing in the perinatal period comes primarily from studies of negative affect and mental illness. From this negative perspective, research has consistently highlighted the importance of subjective wellbeing in the perinatal period. For example, it is estimated that depression and anxiety affect 15–20% of women in the first year after childbirth^10^. While there are less data on fathers, the Paulson and Bazemore^11^ review of depression in fathers estimated that 10% of fathers experienced depression in the perinatal period rising to 25% between 3 and 6 months after birth. Thus, while having a baby is widely assumed to be a happy time for parents these figures highlight the need to know more about the spectrum of wellbeing during this time and how we can best promote subjective wellbeing in the perinatal period for all women and their families.

Alderdice et al.^9^ compared a newly developed Wellbeing in Pregnancy Scale with general wellbeing measures in a sample of 312 pregnant women and found that the mean Satisfaction With Life Scale score was higher than those reported in other studies involving a range of non-pregnant populations^12^. However, the complexity of wellbeing during this time was also evident by how women scored on the WHO5 Wellbeing measure, as the mean score was lower than that found in other studies in the general population^13^. This was mainly related to lower positive responses to two items in five questions, those of ‘I felt active and vigorous’ and ‘I woke up feeling fresh and rested’, which could reflect the physical impact of pregnancy rather than general wellbeing. This also demonstrates the importance of considering the appropriateness of using the above measure in perinatal populations and the importance of further research on the potential impact of the perinatal period on wellbeing, taking into consideration the physical, psychological and social aspects of childbirth and transition to parenthood.

The two focus groups described here where conducted as part of a larger study to develop a pregnancy specific wellbeing measure^9^. This paper aims to describe women’s experiences of subjective wellbeing in the postpartum, using the questions from the European Social Survey Wellbeing module to facilitate discussion, as a first step in exploring appropriate measures of wellbeing for use during this time.

## METHODS

### Design

A qualitative deductive descriptive design was used. Data were collected using focus groups to encourage women to discuss their perceptions of psychological wellbeing and experiences of wellbeing after birth within a group of peers. Directed content analysis was conducted guided by the underlying theory of subjective wellbeing on which the European Social Survey Wellbeing module was based.

### Participants

Nineteen women aged 18–40 years who had given birth within the past 6 months in Northern Ireland participated in two focus groups (n=9 and n=10).

Women were excluded from the study if they did not adequately understand English or had special communication needs. It was essential that women involved in these exploratory discussions were able to understand and fully participate in the ongoing conversation.

### Procedure

Ethical approval and research governance procedures were completed (REC reference number 10/NIR01/24) in advance of women being recruited via local SureStart programmes. An invitation letter and information leaflet were administered through the local centres and SureStart leaders co-ordinated the venue and attendance at the focus group. The focus groups were conducted in a local community centre by a member of the research team (FA). The focus groups lasted approximately 55–75 minutes (the time included information, consent and completion of the European Social Survey Wellbeing questionnaire) and were digitally recorded.

Time was given at the beginning of the session to discuss the information leaflet and women were asked to sign a consent form and complete the sociodemographic questions while sitting together as a group. Participants were then invited to complete the European Social Survey Wellbeing questionnaire and this was used as the basis for discussion. Women were asked about their overall impression of the questionnaire and if they felt the questions were relevant to them, open-ended discussion was facilitated by the content of the questionnaire. Participants kept the questionnaire throughout the discussion but were asked not to disclose their answers unless they wished to do so.

The questionnaire was based on the key items of The European Social Survey Wellbeing module designed by Huppert et al.^14^ and made up of theoretical concepts related to individual wellbeing: emotional wellbeing, satisfying life, vitality, resilience and self-esteem, and positive functioning; and those related to social wellbeing, i.e. supportive relationships, and trust and belonging. The descriptions of the seven components are:

- Emotional wellbeing: The overall balance between the frequency of experiencing positive and negative emotions.
- Satisfying life: Positive evaluation of your life overall.
- Vitality: Having energy, feeling well rested and being physically active.
- Resilience and self-esteem: feeling good about yourself, optimistic for the future and able to deal with difficulties.
- Positive functioning: ‘doing well’ and includes autonomy, competence, engagement, meaning and purpose.
- Supportive Relationships: close relationships with family, friends and others who provide support.
- Trust and belonging: feeling a sense of belonging with support and respect from people where you live.

(Further information can be found at: http://www.europeansocialsurvey.org/index.php?option=com_content&view=article&id=23&Itemid=318)

### Data analysis

Directed content analysis was used, as this study was building on existing subjective wellbeing theory^15^. Transcripts were checked against the audio-recoding, and read and re-read for familiarity. The data were then organized into coding categories that were derived from the seven underlying subjective wellbeing concepts of the European Social Survey Wellbeing questionnaire. Segments of the data were grouped according to each category by identify persistent words and phrases related to the concept^16^ or to specific questions within the questionnaire that reflected the underlying category. Within each category, subcategories were identified, where appropriate, to highlight variations within the category. The principal researcher led the analysis organising the data and developed the interpretation and discussion in each category. Final interpretations were agreed upon by consensus between the authors.

## RESULTS

The sample characteristics of women who participated in the focus groups are given in Table 1. All the women were born in the UK. The majority of women were married and there was a good spread in age and educational qualifications.

**Table 1:** Characteristics of women participating in focus groups (N=19)

*Characteristics*	*n (%)*
**Married or living with partner**	17 (89.5)
**Age** (years)	
≤20	1 (5.3)
21–25	5 (26.3)
26–30	8 (42.1)
31–35	2 (10.5)
36–40	3 (15.8)
>40	0
**Education**	
Up to GCSE or equivalent	11 (58.0)
Up to A Level or equivalent	4 (21.0)
University qualification	4 (21.0)
**Country of birth**	
UK	19 (100)

Overall the response to the questionnaire was positive. Women did not comment on its length despite being a long questionnaire, but in relation to the questions one participant commented: *‘were kind of important questions I thought’* [Focus group 1].

Overall, there was also a recognition that many aspects of wellbeing were compromised after having a baby and so key concepts like autonomy and vitality were difficult to interpret. Women felt that their wellbeing was very much related to their current circumstances and women recognized that the experience of having a baby impacted on their wellbeing but they felt it was important to emphasize this and that it did not mean they had a significant problem or that they were depressed.

‘*I know this is a wellbeing quiz so… … To me, it seems like you’re trying to find out if you have a lot of time for yourself and to sort of nurture yourself, and I don’t basically because I have small children and they get all my time. …I know I can’t be unique in this, so just to be able to be given the opportunity to say, yes, I don’t have “me” time, but it’s not because I’m depressed or…do you know? It’s just because I have too much of a draw on my time. I am a happy enough person, …’* [Focus group 2].

### Emotional wellbeing

The survey questions on emotional wellbeing covered both positive (e.g. happy, enjoyment, calm) and negative emotions (e.g. depression, anxiety). Women talked about having a range of emotions after birth; feeling ‘happy’, ‘exhausted’, ‘stressed’, ‘frightened’, and ‘frustrated’. After the emotional high of giving birth women commented on finding the tiredness and continuous looking-after a new baby stressful.

‘*I got a bit more stressed. Even now, because he’s…he would be still up during the night and stuff, I would be tired, so you end up more stressed and…you lose your temper quick, you know. Even driving in the car, I get terrible road rage, you know, just stupid things. It’s just because you don’t have the same time’* [Focus group 1].

Worrying about their baby was also a source of negative emotions and women expressed varying emotional reactions. Whilst the anxiety of new parenthood was discussed, concerns were not just related to not knowing what to expect with a first child. There were other concerns, for example, not loving a second child as much as the first and looking out for signs of illness.

Woman 1: ‘This is my second child, so I’m a lot more relaxed this time round. I think trying to just stay calm and, you know, things will sort themselves out, because em…you do worry. I know I worried, probably non-stop, with my first child.’

*Woman 2: ‘Mine was the opposite: with my second one, I was worried in case I didn’t love him as much as I love the first’* [Focus group 1].

Participants acknowledged that they tended to discuss the negative emotional aspects of wellbeing rather than the positive. While many anxieties and concerns were provided as examples of emotional wellbeing, the women attending these groups were motivated to get out and meet with other women and engage with each other and their babies. The group discussion flowed with much humour and supportive comments throughout. Women generally found being part of a group a positive emotional experience and shared their experiences readily.

### Satisfaction with life

Overall ‘satisfaction with life’ was not a dominant wellbeing category (Survey question: ‘How satisfied with life as a whole are you’). The focus was more on positive functioning as outlined below. Two women mentioned that they had not planned to have a baby but getting pregnant had changed how they thought about life. Their lives had changed direction and they could no longer relate well to their previous ambitions. Even with the disruption and isolation of not been able to get out of the house as much, these women felt that having a baby positively impacted on their satisfaction with life as a whole.

Woman 1: ‘I always wanted a really good career, like I never thought about children, and then like she was a wee surprise.’

Woman 2: ‘So was he.’

Woman 1: ‘I was just like…why didn’t I ever want kids and what was my life about before?’

*Woman 2: ‘And I don’t understand… Och, I know you do have your days where you want…oh, I want to get out of the house but overall, I think I feel better’* [Focus group1].

### Vitality

Questions on vitality included sleeping well, feeling energized and feeling able to face the challenges that life presents. Not surprisingly, women talked about feeling tired and there was a general acceptance and almost camaraderie around this aspect of postnatal life. Vitality, or the absence of it, was a key concept cutting across other components of wellbeing, impacting on emotional wellbeing and positive functioning.

*‘I would be tired so you end up more stressed’* [Focus group 2].

*‘[when I went home I was] Really exhausted (laughing)! But I had support’* [Focus group 1].

### Resilience and self-esteem

Questions related to resilience and self-esteem included agree/disagree Likert scale responses to ‘At times I feel as if I am a failure’, ‘In general I feel positive about myself’ and ‘When things go wrong in my life, it generally takes me a long time to get back to normal’. Women reported that the time after birth was often challenging in terms of feeling good about themselves. This was attributed to learning new skills, getting to know a new baby and experiencing changes to family dynamics, all of which were challenges to their sense of who they were and their ability to cope.

*‘[you think] …when you get home, are you going to be able to cope on your own and are you doing everything right’* [Focus group 1].

Woman 1: ‘It’s just like I’m not even me anymore sometimes.’

*Woman 2: ‘Yeah. But it does…you can’t…want to sit in all the time with your kids, no matter how much you love them, do you know? Like I have two kids and I absolutely love them to bits, but my first one, I didn’t want to go out – I thought you had to sit in, that you shouldn’t want to leave your child, that you should sit in all the time’* [Focus group 2].

### Positive functioning

As with resilience and self-esteem, the actual sense of ‘doing well’ was also challenging after birth. Questions related to positive functioning again used agree/disagree Likert scales and included: ‘In my daily life I get very little chance to show how capable I am’, ‘Most days I feel a sense of accomplishment from what I do’ and ‘In my daily life I seldom have time to do the things I really enjoy.’

*‘You are doing everything alright, but you just…when you’re in hospital there’s somebody else there to ask for advice, and, when you get home, you sort of panic’* [ Focus group 1].

*‘People underestimate the amount of time you need to get yourself gathered, get out the door’* [Focus group 2]. Women discussed the impact of having a baby on looking after themselves and worried about ‘fitting everything in’. Positive functioning was also impacted upon by a reduction in resources through changes in their work and economic position, while going back to work was discussed with concerns around childcare and getting back into a routine being the main focus.

*‘It’s the worry, when you are on maternity leave, just the way it [money] drops and stuff’* [Focus group 1].

*‘I would put in a lot of extra hours [in work], and I don’t know how I’m going to do that now…I’m always kind of thinking about it’* [Focus group 1].

### Supportive relationships

Questions related to supportive relationships related to having people with whom you can discuss intimate matters, feeling appreciated by those close to you, receiving help and support, and feeling lonely. Woman reported feeling socially isolated after giving birth and highlighted the importance of their relationships in terms of feeling valued and ‘feeling myself’. Changes in relationships were also a source of stress and conflict and these aspects were not adequately picked up by the questionnaire.

#### Partners

Women acknowledged that partners may have found the changes after birth as stressful as they did.

Woman 1: ‘Not just me, my husband didn’t adapt too well to having a baby. I think he thought that this baby should fit in around us, and that our lives will carry on as normal and everything would just be tickety-boo because this baby would just…slot in.’

*Woman 2: ‘Yeah, my husband thinks that too’* [Focus group 2]. The role of partner support in being able to get out or have ‘me’ time was important to women and positive examples were given. The absence of support was perceived to have a significant impact on emotional health after birth. There was discussion with examples being given of women who had not been supported by their partner and experienced depression. Some women felt that a father not bonding with their baby put extra pressure on the mother leading to maternal depression.

*‘But what I found…obviously, everybody’s circumstances are different, but what I found with my husband…well, my fiancé at the time, he was just like another Mummy and I found that – like he was so-hands on with our daughter, you know, I could go out on nights out. He was like – everyone used to say this is like Daddy Daycare – he just took to it like a duck to water. Where, on the other hand, my sister-in-law and her boyfriend, he didn’t take to it like a duck to water, and she suffered from postnatal depression’* [Focus group 2].

#### Mothers/mothers-in-law

Another important supportive relationship was with mothers and their partner’s mother offering much needed support and informal childcare. However, these relationships were at times perceived to be a source of both support and stress.

*‘Now Mummy always offers, and Mummy minds them when I go to work and all’* [Focus group 2].

Woman 1: ‘I think I’ve got closer to my Mummy from he’s been born, really. I see her every day now, and I wouldn’t have saw her as much before. I’m down her house every day or phoning her or texting her, all the time like.’

*Woman 2: ‘My mother-in-law, like from he was born, was always telling me, oh, do this, do that, and you shouldn’t be doing this, and you should be doing that’* [Focus group 2].

### Extended family and friends

Many participants talked about changes to relationships with friends. Comments were made about the difference in relationships with friends who didn’t have children and how friends tended to forget them. There was a sense of loss of friends and a need for their transition to be acknowledged and valued by their friends.

*‘My friends then forgot to ask me to go out, and then I got pregnant again with her and…I was like, oh please, if nobody phones me…I would still hardly get any phone calls now. Because I’ve turned them down so many times, they’re like, “What’s the point in asking [woman’s name], sure she doesn’t go out” ’* [Focus group 2].

However, having children was also an important connection to other women with children. Women talked about the importance of having peers to talk to who had recently been in the same situation. It was important to be able to share experiences and to seek advice from friends and family who had recently been in the same situation.

*‘Yeah, I found…because my sister has a two year old, and my friend has a two year old, but see coming from them, and…I don’t know why or it’s just me, you want their advice, because they’re sort of around the same age, and you know they’re not going to be patronising. They’re just saying, well, like I did this and this is what happened to me.’* [Focus group 2].

### Trust and belonging

There was a mixed response to the trust and belong questions in the questionnaire, some people felt they were very relevant and others that they were not. The questions included responding to the following statements: ‘Most people can be trusted’, ‘I feel close to the people in my local area’, and ‘Most of the time people are helpful’. Women reported both positive and negative experiences of belonging to their local community.

Woman 1: ‘But see the question about your local area, I’ve been living in my area for nearly five years, and I couldn’t tell you my next door neighbour’s name.’

Woman 2: ‘The street I live in, like they’re brilliant, they’re really, really good, but [they’re all older] people like that as well and…but they just kind of absorbed me into it, which is great.’

*Woman 3: ‘I walked in here today and I’ve lived on my street four years, and someone said to me “You live up the street from me.”. I haven’t seen her before, ever, and she only knew me because I – she used to be walking past with the pram’* [Focus group 2].

While women had varying experiences with their local community, they found it helpful to meet with other women from their local community to compare stories and felt that social support in the community was important.

## DISCUSSION

Overall women were open to the concept of subjective wellbeing and could relate to the many aspects that were explored. However, they also felt that their wellbeing was impacted on by their current circumstances and that variations from how they usually felt and thought should not necessarily be considered a sign of a problem. This is in keeping with WHO-5 wellbeing scores for women during pregnancy that have been found to be reduced in certain aspects of wellbeing, e.g. vitality, that may be related to symptoms of pregnancy rather than a perceived reduction in wellbeing^9^.

Subjective wellbeing is a particularly attractive concept within the context of maternity care because the majority of women are well and the priority is to maximize the benefits for each woman and her family during this important time rather than simply aiming to prevent ill health. The concept of wellbeing fits well with the ethos of maternity care and provides a valuable framework for professional discussions around what women need physically, psychologically and socially after the birth of their baby. The underlying concepts of subjective wellbeing go beyond an assessment of mood, for example recognizing the changes in different relationships and identifying the sources of isolation that can be challenging.

In terms of the concepts that make up subjective wellbeing, satisfaction with life is perceived to be relatively stable over one’s lifetime but is thought to be sensitive to major life events such as childbearing^17^. In our study the satisfaction with the life component of wellbeing was reported by some women to be a positive consequence of having a baby, although women were generally not interested in discussing this in detail. Positive perspectives on satisfaction with life are in keeping with other studies on pregnancy and postpartum that suggest that women have a high satisfaction with their life at this time^9,18,19^.

Women in the focus groups struggled with the questions related to trust and belonging to their local community with women reporting that they did not feel engaged with their local community and neighbours. They did, however, value meeting up with women in their local community who had babies. The value of informal community support for women is also reflected in research on the general population that suggests that informal community socializing is strongly associated with higher life satisfaction in women, irrespective of parenting status^20^. These finding suggest that providing women with opportunities to meet other women in their local community, either through support groups or activity-based groups such as baby yoga or walking groups, are appropriate mechanisms to help manage the often-reported experiences of social isolation after birth^18^.

A key category for women in the focus groups was supportive relationships, particularly with partners. In the general population women’s wellbeing has been found to be more reliant on positive inter-personal relations than for men^21^. Within the perinatal period, Gebuza et al.^18^ found that social support after birth, particularly instrumental support, predicted a woman’s satisfaction with life. A large cohort study in Norway found that the relationship with the partner was particularly important for optimizing wellbeing around the time of birth^19^. Our study findings are in keeping with these findings. In addition to support in the immediate postpartum period, women expressed concerns about going back to work, the changes they would face and the impact it would have on them and their family. Mistry et al.^22^ suggest that emotional or functional support, spending time with their children, and difficulty paying for childcare, were all significantly associated with reduced wellbeing.

This is a vulnerable time for women and it is important that we have a better understanding about the spectrum of wellbeing and what support women need. While women in this study did not want the reported lowering in their wellbeing to be interpreted as ‘being depressed’, they equally recognized that women could experience mental illness particularly if unsupported. There was also an acknowledgement by women that their partners may also have problems adapting after the birth. As with current literature on depression, this highlights the need to identify and support women and their partners who are struggling psychologically^10,11^.

In this study we used the European Social Survey Wellbeing module questionnaire to explore subjective wellbeing, however there are a number of standardized wellbeing measures that have been developed for use in the general population that could be used in the perinatal period^13,23^. Our findings suggest that caution is necessary in interpreting some aspects of subjective wellbeing, such as vitality, which will be reduced because of the normal consequences of pregnancy and having a baby. Also consideration should be given to developing a perinatal specific measure as there was evidence of challenges in supportive relationships and positive functioning, that may not be reflected in general measures.

The comprehensive framework of subjective wellbeing used in the present study will allow assessment of a number of domains that women have highlighted as important to them. Although women in this study related to the underlying concepts of subjective wellbeing they perceived their wellbeing to be different after birth and it is important that psychometric data are developed for general measures during the postnatal period to identify variations in wellbeing from the general population.

The use of questionnaires and validated wellbeing scales may provide an alternative approach to current approaches in identifying perinatal psychological problems. Comparison with measures currently used in practice, such as the Edinburgh Postnatal Depression Scale, would further elucidate the relationship between positive and negative aspects of wellbeing. Measuring wellbeing at this time would help women to discuss mental health in a broader spectrum of positive and negative emotions that may help to destigmatize mental illness. However, the benefits of these measures require more research before implementation in practice. In addition to instrument development, further qualitative research that follows women through the first year postpartum would provide important insights into changes in subjective wellbeing at key transitions, including infant feeding, returning to work and planning future pregnancies.

### Limitations and strengths

This study explored subjective wellbeing using a detailed theoretical based questionnaire that provided insights into the many facets of subjective wellbeing and how these might be impacted upon during the transition to parenthood. However, building on existing theory on subjective wellbeing to guide the research also has some limitations in that researchers approach the data with an informed bias^15^. The study was limited by its size, however there was a good range across age and education groups of women who participated.

## CONCLUSIONS

Women perceive their subjective wellbeing to be different after birth with social relationships and positive functioning potential challenges. Assessment of subjective wellbeing after birth could provide a better understanding of the aspects of challenged wellbeing and facilitate the provision of tailored support for women and their families to maximize their ongoing better health and wellbeing.

## References

[1] World Health Organisation (2012). Measurement of and target-setting for well-being: an initiative by the WHO Regional Office for Europe.

[2] Michaelson J, Abdallah S, Steuer N, Thompson S (2009). National Accounts of Wellbeing: bringing real wealth onto the balance sheet 2009.

[3] Deiner E, Chan MY (2011). Happy People Live Longer: Subjective Wellbeing Contributes to Health and Longevity. Applied Psychology: Health and Wellbeing.

[4] Department of Health (2014). Wellbeing across the lifecourse: The relationship between wellbeing and health.

[5] Jomeen J (2004). The importance of assessing psychological status during pregnancy, childbirth and the postnatal period as a multidimensional construct: A literature review. Clin Eff Nurs.

[6] Royal College of Midwives (2012). Maternal Emotional Wellbeing and Infant Development: A Good Practice Guide for Midwives.

[7] Myrskylä M, Margolis R (2014). Happiness: before and after the kids. Demography.

[8] Aassve A, Goisis A, Sironi M (2012). Happiness and Childbearing Across Europe. Soc Indic Res.

[9] Alderdice F, McNeill J, Gargan P, Perra O (2017). Preliminary evaluation of the Wellbeing in Pregnancy (WiP) Questionnaire. J Psychosom Obstet Gynaecol.

[10] National Institute for Health and Care Excellence (2014). Antenatal and postnatal mental health guidelines. Clinical Guidelines CG192.

[11] Paulson JF, Bazemore SD (2010). Prenatal and postpartum depression in fathers and its association with maternal depression: a meta-analysis. JAMA.

[12] Pavot W, Diener E, Diener E (2009). Review of the satisfaction with life scale. Assessing Well-Being. Social Indicators Research Series.

[13] Bech P (2004). Measuring the dimension of psychological general wellbeing by the WHO-5. Qual Life Newslett.

[14] Huppert FA, Marks N, Clark A (2009). Measuring wellbeing across Europe: Description of the ESS Wellbeing Module and preliminary findings. Soc Indic Res.

[15] Hsieh HF, Shannon SE (2005). Three Approaches to Qualitative Content Analysis. Qual Health Res.

[16] Morse JM, Field PA (1995). Qualitative Research Methods for Health Professionals.

[17] Aasheim V, Waldenström U, Rasmussen S, Espehaug B, Schytt E (2014). Satisfaction with life during pregnancy and early motherhood in first-time mothers of advanced age: a population-based longitudinal study. BMC Pregnancy Childbirth.

[18] Gebuza G, Kazmierczak M, Mieczkowska E, Gierszewska M, Kotzbach R (2014). Life Satisfaction and social support received by women in the perinatal period. Adv Clin Exp Med.

[19] Dyrdal GM, Roysamb E, Bang Nes R, Vitterso J (2011). Can a happy relationship predict a happy life? A population-based study of maternal well-being during the life transiton of pregnancy, infancy and toddlerhood. J of Happiness Stud.

[20] Kroll C (2011). Different things make different people happy: examining social capital and subjective wellbeing by gender and parental status. Soc Indic Res.

[21] Pinquat M, Sorensen S (2001). Gender differences in self-concept and psychological wellbeing in old age: a meta-analysis. J Gerontol B Psychol Sci Soc Sci.

[22] Mistry R, Stevens GD, Sareen H, De Vogli R, Halfon N (2007). Parenting Related Stressors and Self-Reported Mental health of Mothers with Young Children. Am J Public Health.

[23] Tennant R, Hiller L, Fishwick R (2007). The Warwick-Edinburgh Mental Wellbeing Scale (WEMWBS): Development and UK validation. Health Qual Life Outcomes.

